# IL-23 Enhances C-Fiber-Mediated and Blue Light-Induced Spontaneous Pain in Female Mice

**DOI:** 10.3389/fimmu.2021.787565

**Published:** 2021-12-07

**Authors:** Jasmine Ji, Qianru He, Xin Luo, Sangsu Bang, Yutaka Matsuoka, Aidan McGinnis, Andrea G. Nackley, Ru-Rong Ji

**Affiliations:** ^1^ Center for Translational Pain Medicine, Department of Anesthesiology, Duke University Medical Center, Durham, NC, United States; ^2^ Neuroscience Department, Wellesley College, Wellesley, MA, United States; ^3^ Department of Pharmacology and Cancer Biology, Duke University Medical Center, Durham, NC, United States; ^4^ Department of Cell Biology, Duke University Medical Center, Durham, NC, United States; ^5^ Department of Neurobiology, Duke University Medical Center, Durham, NC, United States

**Keywords:** dorsal root ganglion, IL-23, macrophage, mechanical allodynia, nociceptor, optogenetics, spontaneous pain, sex dimorphism, TRPV1

## Abstract

The incidence of chronic pain is especially high in women, but the underlying mechanisms remain poorly understood. Interleukin-23 (IL-23) is a pro-inflammatory cytokine and contributes to inflammatory diseases (e.g., arthritis and psoriasis) through dendritic/T cell signaling. Here we examined the IL-23 involvement in sexual dimorphism of pain, using an optogenetic approach in transgenic mice expressing channelrhodopsin-2 (ChR2) in TRPV1-positive nociceptive neurons. *In situ* hybridization revealed that compared to males, females had a significantly larger portion of small-sized (100-200 μm^2^) *Trpv1* ^+^ neurons in dorsal root ganglion (DRG). Blue light stimulation of a hindpaw of transgenic mice induced intensity-dependent spontaneous pain. At the highest intensity, females showed more intense spontaneous pain than males. Intraplantar injection of IL-23 (100 ng) induced mechanical allodynia in females only but had no effects on paw edema. Furthermore, intraplantar IL-23 only potentiated blue light-induced pain in females, and intrathecal injection of IL-23 also potentiated low-dose capsaicin (500 ng) induced spontaneous pain in females but not males. IL-23 expresses in DRG macrophages of both sexes. Intrathecal injection of IL-23 induced significantly greater p38 phosphorylation (p-p38), a marker of nociceptor activation, in DRGs of female mice than male mice. In THP-1 human macrophages estrogen and chemotherapy co-application increased IL-23 secretion, and furthermore, estrogen and IL-23 co-application, but not estrogen and IL-23 alone, significantly increased IL-17A release. These findings suggest a novel role of IL-23 in macrophage signaling and female-dominant pain, including C-fiber-mediated spontaneous pain. Our study has also provided new insight into cytokine-mediated macrophage-nociceptor interactions, in a sex-dependent manner.

## Introduction

The majority of primary sensory neurons in dorsal root ganglia (DRG) and trigeminal ganglia are nociceptors, representing a heterogeneous population of unmyelinated C-fibers and myelinated Aδ fibers (1–3). Transient receptor potential vanilloid type 1 (TRPV1) is one of the best markers for C-fiber nociceptors and integrates thermal, mechanical, and noxious stimuli (4, 5), as well as infections and danger-associated molecular patterns (6, 7), into pain signals. Inflammation produces inflammatory mediators, such as prostaglandins, ATP, and bradykinin, which evoke spontaneous pain and produce nociceptor sensitization (peripheral sensitization) that can trigger pain hypersensitivity *via* TRP channels, such as TRPV1, TRPA1, and TRPV4, and sodium channels, such as Nav1.7 and Nav1.8 (2, 8). It is emerging as a hot topic to investigate interactions between immune cells, such as macrophages, neutrophils, dendritic cells, stem cells, and T cells and nociceptors in the context of inflammation and pain (9–12). Studies suggest that immune cell-derived pro-inflammatory cytokines (e.g., TNF-α, IL-1β, IL-17) and pro-inflammatory chemokines (e.g., CCL2 and CXCL1) can act directly on nociceptors to elicit peripheral sensitization and pain (13–16). However, the interactions between nociceptors and cytokines/chemokines are not fully understood. For example, CXCL5 is highly induced by ultraviolet B (UVB) and mediates UVB irradiation-induced pain. Notably, CXCL5 has no direct action on nociceptors. Instead, CXCL5 indirectly activates nociceptors *via* macrophage signaling (17).

Interleukin 23 (IL-23) is a pro-inflammatory cytokine of the interleukin 12 (IL-12) family (18), released by dendritic cells, and exerts its functions through the IL-23 receptor (IL-23R) in T cells (e.g., Th17 cells). IL-23 regulates inflammatory diseases such as psoriasis and osteoarthritis (19), as well as arthritic pain (20) and histamine-evoked itch (21). Interestingly, C-fiber nociceptor has been shown to activate the local IL-23/IL-17 cascade in the skin *via* release of neuropeptide calcitonin gene-related peptide (CGRP) in a murine model of psoriasis, induced by TLR7 agonist imiquimod (19, 22). Our early study demonstrated that imiquimod can also act on nociceptor TLR7 to elicit pruritus (23), a remarkable clinical feature of psoriasis. Nociceptive sensory fibers can drive IL-23 production from CD301b+ dermal dendritic cells, leading to protective cutaneous immunity (24). Furthermore, Cohen et al. demonstrated that TRPV1+ neuron activation is sufficient for host defense against infections (C. albicans and S. aureus), by inducing local type 17 immune response (25). However, IL-23 signaling in macrophages and DRG, where nociceptor cell bodies are localized (3), is largely known.

Notably, IL-23 also modulates macrophage phenotype and elicits macrophage IL-17 production (26). In our recent study (27), we investigated how peripheral IL-23 signaling modulates pain in male and female mice. We demonstrated that IL-23 is both sufficient and required to evoke mechanical pain (mechanical allodynia) in female mice *via* IL-23R signaling. Intriguingly, IL-23 cannot directly activate nociceptors. Instead, IL-23 triggers macrophage release of IL-17A, which activates the IL-17A receptor (IL-17RA) in nociceptors to elicit mechanical pain in females *via* TRPV1 (27). However, it is not fully understood how IL-23 modulates nociceptor activity and drives different pain modalities, including spontaneous pain in a sex-dependent manner. In this follow-up study, we employed an optogenetic approach to investigate light-induced pain *via* activation of TRPV1-expressing nociceptors and the impact of IL-23 on such pain and its sex dimorphism. We also characterized *Trpv1* expression in nociceptors grouped by various size ranges in both sexes. We further investigated the mechanisms by which IL-23 and IL-17 are released in human macrophages. Our results provide new insights into cytokine-nociceptor signaling in a sex-dependent manner.

## Materials and Methods

### Animals

Adult *Trpv1*-Cre mice (JAX stock #017769) and ChR2-EYFP-report mice (JAX stock # 012569, Ai32 mice) (28), with C57BL/6 background (8-12 weeks, 25-30 g) of both sexes, were purchased from Jackson laboratories. These two lines were crossed to obtain TRPV1-ChR2 mice, wherein ChR2 is expressed in TRPV1-expressing nociceptors (29). Mice were maintained at the Animal Care Facilities of the Duke University School of Medicine. All animals were kept on a 12 hour light/dark cycle and provided with food and water ad libitum. 2-5 animals were housed per cage at ambient temperature. All animal procedures were approved by the Institutional Animal Care and Use Committee (IACUC) of Duke University. All animal experiments were conducted according to the National Institutes of Health Guide for the Care and Use of Laboratory Animals.

### Reagents and Injections

Recombinant mouse IL-23 protein was purchased from Biolegend (Cat# 589004). Capsaicin was from Sigma-Aldrich (Cat# M2028). We obtained 17β-Estradiol (Estrogen) from Cayman Chemical (Cat# 10006315) and paclitaxel from Sigma-Aldrich (Cat# T7191). IL-23 injection contained 100 ng of IL-23 in 10 µL vehicle (PBS). Prior to IL-23 or vehicle injections, animals were briefly anesthetized with 4% isoflurane. Intraplantar injection was administered to the left hindpaw using a 30-gauge needle with a microsyringe. Spinal cord puncture for intrathecal injection was administered into cerebrospinal fluid in the region between L5 and L6 (30).

### Optogenetic Stimulation

Two days prior to testing, animals were given an hour to habituate to the testing environment. Optogenetic stimulation was performed with a 470 nm LED (Thor Labs, M470F3) connected to a 1000 µm fiber (Prizmatix) as well as an LED driver (D2200, Thor Labs), used to adjust stimulus parameters. A 10 Hz blue light was used as previously described (28). Stimulation was performed by holding the 1000 µm fiber 1-2 mm below the plantar region of the left hindpaw for 20 seconds without making contact. Animal behavior was recorded using an iPhone 5S camera (30 frames/second). A resting period of 10 minutes was allotted between each of three trials. Naive animals of both sexes were stimulated at intensities of 2 mW/mm^2^, 4 mW/mm^2^, and 6 mW/mm^2^ (three trials/intensity) to establish an intensity-dependence of blue light-induced pain. Naive animals of both sexes were stimulated with blue length at a low intensity (2 mW/mm^2^) prior to IL-23 injection and again one hour following IL-23 injection to study the effects of IL-23 on blue light-induced pain. The experimenter was blind to the treatment (vehicle *vs*. drug). Duration of pain was measured as the time in seconds animals spent lifting and/or lifting their afflicted paw (28).

### Von Frey Up-Down Testing for Mechanical Pain

Two days prior to testing, animals were given an hour to habituate to the testing environment. Mechanical threshold was assessed using Dixon’s Up-Down method (31), with calibrated von Frey filaments (0.16 g, 0.4 g, 0.6 g, 1.0 g, 2.0 g) (North Coast Medical). Filaments were applied to the plantar region of the left hindpaw, beginning with the lowest filament (0.16 g). If pain behavior was observed, an “X” was recorded and a lower filament was used (0.16 g remained at 0.16 g). If pain behavior was not observed, an “O” was recorded and a higher filament was used. Each animal was stimulated six times at each of three time points (baseline, one hour, and three hours following injection). The order of Os and Xs recorded were then converted to mechanical thresholds (in grams).

### *In Situ* Hybridization and Quantification

Animals (5 males and 5 females) were briefly anesthetized with 4% isoflurane, then perfused through the left ventricle PBS and 4% PFA using 30-mL syringes. Following perfusion, L4 and L5 DRGs were collected and postfixed in 4% formaldehyde at 4°C for 2 hours. DRGs were placed in a 30% sucrose solution and dehydrated overnight. DRG tissues were then embedded in OCT medium (Tissue-Tek), sectioned (14 μm) in a cryostat, and thaw-mounted onto Superfrost Plus slides (VWR). *In situ* hybridization was performed using the RNAscope system (Advanced Cell Diagnostics) following the manufacturer’s instructions. Probe for murine *Trpv-1* (313331-C2) was applied in this study. DRG sections were dehydrated by washing in PBS (2 x 5 min) and placing in an increasing alcohol gradient, then pre-treated with hydrogen peroxide, washed, and pre-treated with Protease IV. The Trpv1 probe (mixed with RNAscope Diluent, 1:50) was added to the sections, which were heated to 40°C for 2 hours, then washed with wash buffer (2 x 2 min). The probe was amplified using Amp 1, Amp 2, and Amp 3 solution, with washing after each addition. HRP-C2 was then added to the sections. After washing with wash buffer, Fluorescein-C2 was added (CY5, 1:750), and the sections were again heated to 40°C for 30 min. Following additional washing, HRP blocker was added to the sections, which were heated to 40°C for a third time for 15 min. Slides were again washed with wash buffer, then mounted with mounting media. All images were acquired with the same settings using a Nikon fluorescence microscope under 20x magnification. We selected four non-adjacent DRG sections from each animal and included five animals per sex for data analysis. The sizes of *Trpv1*-positive neurons were analyzed blindly using Image J.

### Immunohistochemistry and Quantification

Immunohistochemistry of IL-23 and macrophage marker F4/80 was performed on naïve animals of both sexes. Immunohistochemistry of P-p38 was performed on animals of both sexes who had undergone intrathecal injection of vehicle or IL-23. Thirty minutes after injection, animals were anesthetized with isoflurane and perfused through the left ventricle with first PBS, then 4% formaldehyde. After perfusion, the L4-L5 DRGs were collected and postfixed in 4% formaldehyde at 4°C for 2 hours. The DRGs were then placed in a solution of 30% sucrose in PBS at 4°C and dehydrated overnight. The DRGs were mounted with optimal cutting temperature medium (Tissue-Tek), then cut with a cryostat (Leica) to a thickness of 14 μm and thaw-mounted onto Superfrost Plus slides (VWR). The sections were blocked with blocking buffer (5% donkey serum and 0.1% Triton X-100 in PBS) for 1 hour at room temperature. Afterward, primary antibodies were diluted in 1% BSA and 0.2% Triton X-100; the sections were then incubated with the primary antibodies overnight at 4°C. The primary antibodies include IL-23 monoclonal antibody (mouse, 1:500, Santa Cruz, sc-271219), F4/80 monoclonal antibody (rat, 1:100, Invitrogen, PA5-21399), and p-p38 (rabbit, 1:500, Cell Signaling, #9212). For double-staining of IL-23 and F4/80, two primary antibodies from different species were mixed. Following incubation, the sections were washed with PBS (3 x 5 min), and incubated with secondary antibodies (1:500, Jackson ImmunoResearch) for 1-2 at room temperature. The secondary antibodies include 488-conjugated anti-mouse, cy3-conjugated anti-rat, and cy3-conjugated anti-rabbit antibodies. For double staining, two secondary antibodies were also mixed. After washing with PBS again (3 x 5 min), the sections were mounted with a coverslip and the mounting medium, Prolong Gold (Life Technologies), and allowed to dry overnight at room temperature. Images were obtained using a Leica Confocal Microscope at the Duke University Microscope Center. The number of IL-23 and F4/80 double-positive cells in 4 DRG sections per animal was quantified blindly and calculated as the number of cells per mm^2^ of tissue.

### ELISA Analysis in THP-1 Human Macrophages

THP-1 cells (human macrophage cell line) were purchased from the Duke core facility (ATCC # TIP202). Cells were cultured in high glucose (4.5 g/L) RPMI 1640 Medium containing 10% (v/v) FBS at 37°C. THP-1 cells were treated in RPMI media containing 250 nM PMA (Sigma) for 48 hours for differentiation and then washed with PBS to eliminate non-adherent cells. The adherent cells were differentiated (mature) human macrophages. To examine IL-23 and IL-17A secretion in THP1 cells, we purchased hIL-23 ELISA kit from Biolegend (# 437607, San Diego, CA) and hIL-17A ELISA kit from Meso Scale Diagnostics (# K151RFD-1, Rockville, MD). After seeding matured THP1 cells (500,000, 400 μl), we treated these macrophages with β-estradiol (1 ng/ml), paclitaxel (1 µg/ml), and their combination for 24 hours at 37°C. We collected 200 μl of the culture media for ELISA assays. We measured secreted cytokine levels using a Bio-Rad plate reader for hIL-23 and a MESO QuickPlex SQ 120 for hIL-17A, according to the manufacturer’s protocol.

### Statistics

All data in this study are expressed as mean ± SEM. All sample sizes are included in figure legends. Animals were randomly assigned to each experimental group. The results of the behavioral tests were analyzed with two-way ANOVA, or one-way ANOVA, followed by Bonferroni’s *post-hoc* test, or by unpaired Student’s t-test. In cases when the data appeared to be non-normally distributed, a normality test was performed (D’Agostino-Pearson omnibus normality test), followed by a non-parametric Mann-Whitney test or Kruskal-Wallis test. Differences between groups were deemed statistically significant if the *p*-value was less than 0.05. Statistical significance was labeled as *p < 0.05, **p < 0.01, ***p < 0.001, and ****p < 0.0001.

## Results

### Female and Male Mice Exhibit Distinct Size Distributions of TRPV1-Positive Neurons

**Figure 1:**
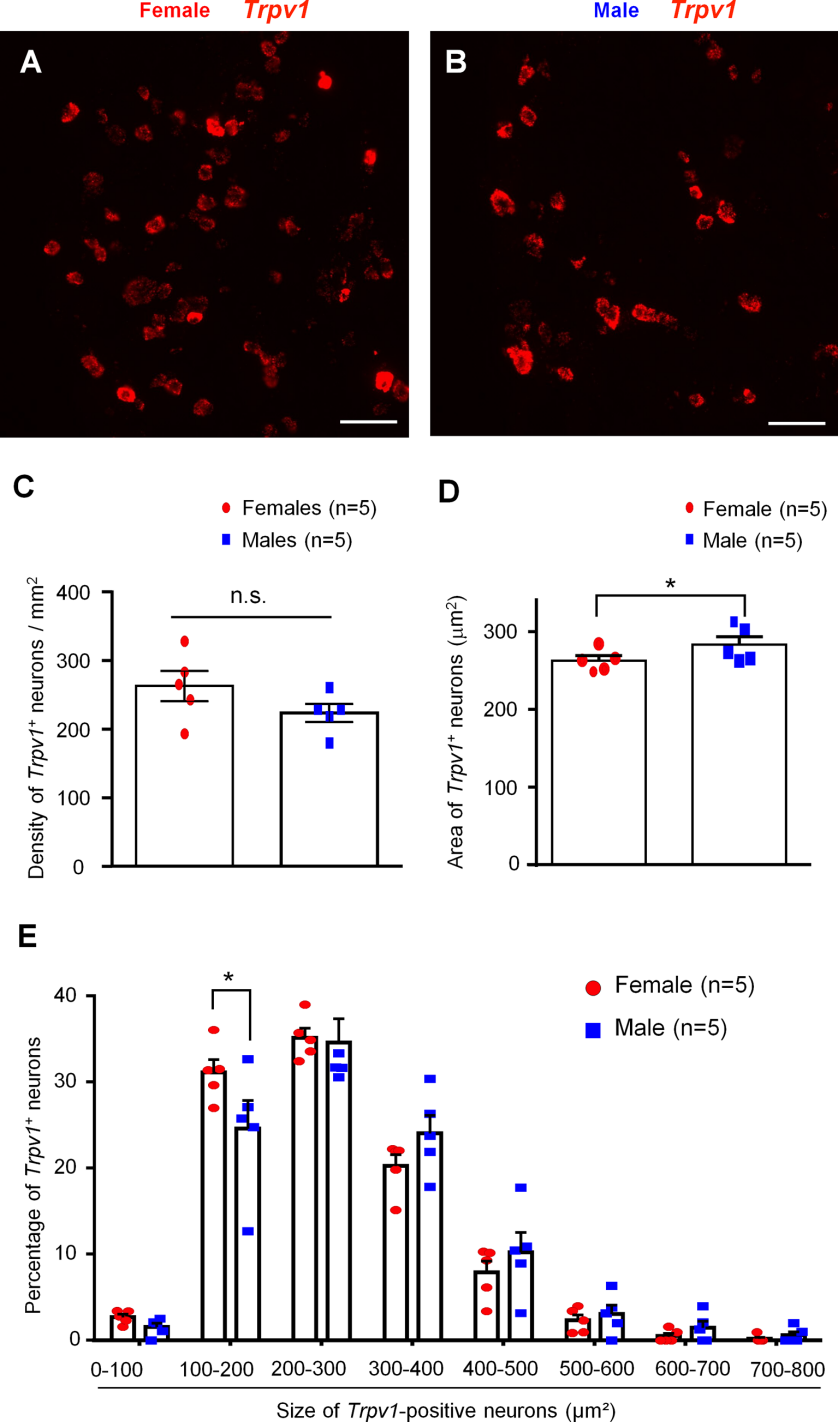
*In situ* hybridization showing distinct *Tprv1* mRNA expression in mouse DRG of both sexes. **(A, B)** RNAscope was performed on DRG sections of female **(A)** and male **(B)** mice to assess the relative number of *Trpv1* mRNA-positive neurons. Imaging was performed with a fluorescence microscope. Scale bars, 75 μm. **(C, D)** Density **(C)** and area **(D)** of *Trpv1* ^+^ neurons in DRG sections of both sexes. RNAscope results were quantified with Image J and analyzed with GraphPad Prism using an unpaired t-test. *p < 0.05. n.s., not significant. **(E)** Size distribution frequency of *Trpv1* ^+^ neurons in DRG of mice of both sexes. Percentages represent number of *Trpv1* ^+^ neurons within size range over total number of the positive neurons for the specified sex. *p < 0.05, unpaired t-test. Data shown as mean ± SEM. n = 5 mice/group per sex. For each animal, 4 DRG sections were analyzed.

Our recent study showed comparable expression of *Trpv1* mRNA in male and female mice (Luo et al., 2021). Given the important role of TRPV1 in immune cells and nociceptor interactions, we conducted additional analysis of *Trpv1* expression using a cohort of 10 male and female mice (n = 5 for each sex). We used a highly selective and sensitive RNAscope protocol (Advanced Cell Diagnostics) to assess the relative numbers of TRPV1^+^) neurons in the DRG of mice of both sexes (**Figures 1A, B** **)**. We found that females appear to have a slightly greater number of *Trpv1* ^+^ neurons than males, albeit not to a significant extent (female: 30.70 ± 3.48; male: 24.90 ± 1.55, per arbitrary unit, *p* = 0.1825, n = 5 mice/sex) (**Figure 1C**). We also found that the average size of *Trpv1* ^+^ neurons was slightly but significantly smaller in female mice *vs*. male mice (female: 262.68 ± 6.28 µm^2^; male: 283.21 ± 10.22 µm^2^, *p* < 0.05, n = 5 mice/sex) (**Figure 1D**). We then assessed the sizes of the *Trpv1* ^+^ neurons in both sexes, organizing the neurons into size categories of 0-100 µm^2^, 100-200 µm^2^, 200-300 µm^2^, up to 700-800 µm^2^ (**Figure 1E**). To account for the difference in the number of *Trpv1* ^+^ neurons for each sex, we expressed our quantifications as percentages, calculated as the number of *Trpv1* ^+^ neurons in the size category for the specified sex over the total number of *Trpv1* ^+^-positive neurons for the specified sex. Strikingly, females had a significantly higher percentage than males in the size category of 100-200 µm^2^ (female: 31.11 ± 1.48%, male 24.57 ± 3.28%, *p* < 0.05, n = 5 mice/sex, **Figure 1E**). However, there was no significant difference between females and males for the other size categories, although males appeared to have larger populations in the 300-400 and 400-500 µm^2^ size ranges (**Figure 1E**). Collectively, these results suggest that *Trpv1* ^+^ may be present in more small-sized nociceptive neurons in females compared to males.

### IL-23 Potentiates Blue Light-Induced Pain in Female but Not Male Mice

**Figure 2:**
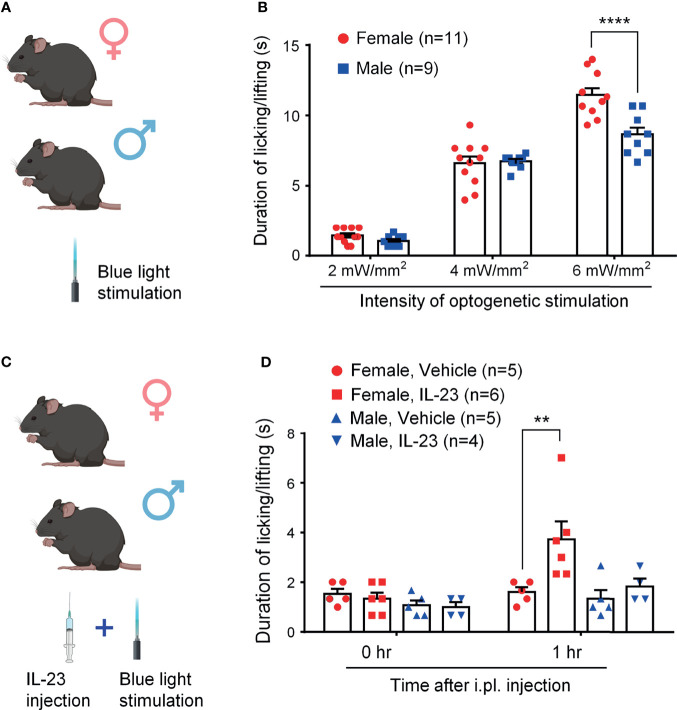
IL-23 potentiates blue light-induced, intensity-dependent, and sex-dependent spontaneous pain in ChR2-TRPV1 reporter mice. **(A, C)** Schematic of experimental design. Mice of both sexes were treated either with blue light only **(A)** or with IL-23 injection followed by blue light stimulation **(C)**. Light-induced spontaneous pain was assessed by duration of pain response. **(B)** Blue light-induced spontaneous pain in male and female mice, as assessed by time spent on licking and lifting behavior. Optogenetic stimulation was applied to the left hindpaw plantar surface for 20 seconds. Duration of pain response was timed in seconds. n = 9 (male) or 11 (female) mice/group. ****p < 0.0001, Two-way ANOVA followed by Bonferroni *post-hoc* test. **(D)** Effects of IL-23 on blue light-induced pain in both sexes. One hour prior to optogenetic stimulation, mice were given an intraplantar injection of either vehicle (PBS, 10 μL) or IL-23 (100 ng, 10 μL). Optogenetic stimulation was applied to the left hindpaw plantar surface for 20 seconds at 2 mW/mm^2^. n = 4-6 mice/group. **p < 0.01, Two-way ANOVA followed by Bonferroni posthoc test. Data shown as mean ± SEM. Illustrations in A and C were made with BioRender with license.

Optogenetic approaches have been used to activate or suppress nociceptors for pain modulation (29, 32). However, sex-dimorphism of light-stimulated pain has not been investigated. We first tested blue light-induced pain in both sexes by stimulating TRPV1-ChR2 mice at three different intensities (2 mW/mm^2^, 4 mW/mm^2^, and 6 mW/mm^2^) and measuring the duration of the resulting pain behavior (28, 29). **Figure 2A** shows the experimental paradigm of light stimulation. We found that blue light-induced spontaneous pain is intensity-dependent: mice of both sexes showed a significantly higher duration of pain response with each increase in intensity (*P <*0.0001, **Figure 2B**). We did not observe sex differences in light-induced pain at the intensities of 2 mW/mm^2^ and 4 mW/mm^2^ (*p* > 0.05, **Figure 2B**). Interestingly, females showed a significantly higher duration of pain response than males at the highest intensity (6 mW/mm^2^, *p* < 0.0001, **Figure 2B**).

We then examined whether IL-23 could potentiate blue light-induced pain in mice of both sexes (**Figure 2C**). To see an additive effect of two treatments, we chose a low intensity of light stimulation (2 mW/mm^2^). We found that while durations of pain response were comparable for mice of both sexes at baseline, females demonstrated significantly higher durations of pain response at one hour following intraplantar IL-23 injection (100 ng), whereas males did not (**Figure 2D**). These findings suggest that IL-23 potentiates blue light-induced pain in only female mice.

### IL-23 Induces Mechanical Allodynia in Female Mice but Not Males

**Figure 3:**
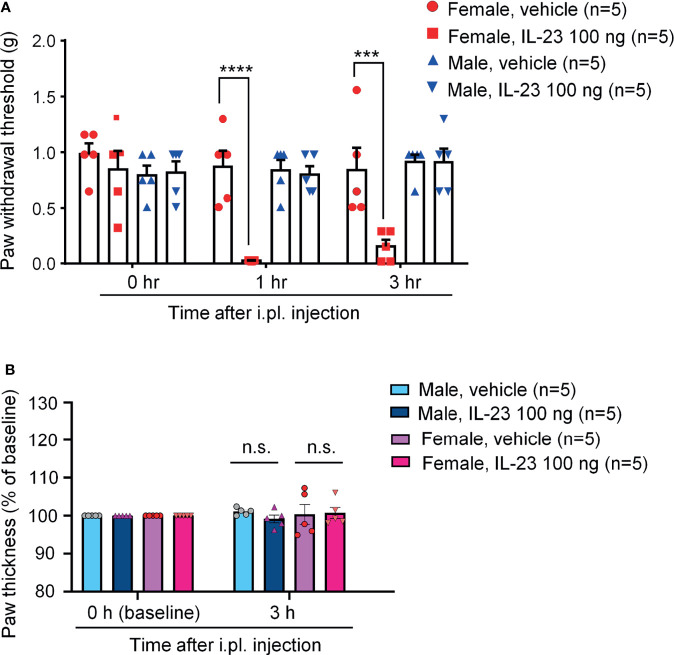
Intraplantar IL-23 injection induces mechanical allodynia only in female mice without affecting paw edema. **(A)** Mechanical sensitivity in males and females. Paw withdrawal threshold was determined using the von Frey Up-Down method at baseline, 1 hour after injection, and 3 hours after injection. **(B)** Paw thickness in males and females 3 hours after IL-23 or vehicle injection. Data shown as mean ± SEM. n = 5 mice/group. ***p < 0.001, ****p < 0.0001, Two-way ANOVA followed by Bonferroni posthoc test. n.s., not significant. Mice were given an intraplantar injection of either vehicle (PBS, 10 μL) or IL-23 (100 ng, 10 μL).

Our recent study demonstrated that IL-23 was sufficient to induce mechanical pain in female but not male mice (27). We found that intraplantar IL-23 (100 ng) could induce lasting mechanical pain (over 5 hours) in females, but not in males, using von Frey testing. To further understand the role of IL-23 in the sex dimorphism of mechanical pain and to confirm the findings of Luo et al., we used the von-Frey Up-Down test to assess the withdrawal thresholds of mice of both sexes at baseline, one hour, and three hours after intraplantar IL-23 injection (100 ng). We found that while the withdrawal thresholds of males injected with IL-23 did not change significantly, the withdrawal thresholds of females injected with IL-23 decreased significantly one hour and three hours following injection. Females injected with IL-23 had significantly lower paw withdrawal thresholds than their vehicle counterparts at both time points (*p* < 0.001 at 1 h at 3 h, **Figure 3A**). Our finding confirms that IL-23 does participate in the sex dimorphism of mechanical pain.

Intense and persistent activation of C-fibers, such as TRPV1 and TRPA1 agonists, is known to produce remarkable edema and neurogenic inflammation (33, 34). Our recent study shows necessity of TRPV1 for IL-23-induced mechanical pain in females (27). To this end, we measured paw thickness 3 hours after intraplantar injection of IL-23 (100 ng) or vehicle (PBS) in male and female mice. We found no difference in paw thickness in vehicle and IL-23 injected paws in both sexes (**Figure 3B**). This finding suggested that IL-23 does not induce edema, indicating different mechanisms of TRPV1 activation by capsaicin (direct mechanism) *vs*. IL-23 (indirect mechanism).

### IL-23 Potentiates Capsaicin-Induced Spontaneous Pain in Female Mice but Not Males

**Figure 4:**
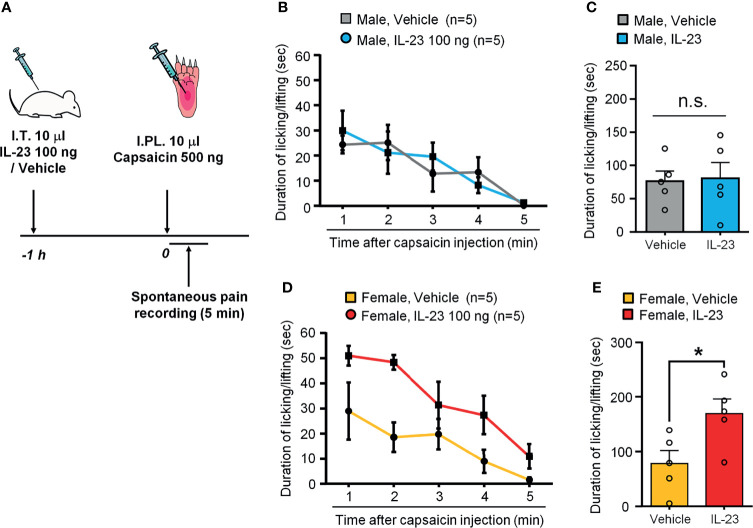
IL-23 potentiates capsaicin-induced spontaneous pain in females. **(A)** Schematic of experimental design. Mice of either sex were given an intrathecal injection of either vehicle (PBS) or IL-23 (100 ng, 10 µL) 1 hour prior to intraplantar injection of capsaicin (500 ng, 10 µL). Pain was assessed by duration of pain response (in seconds) over five minutes following capsaicin injection. **(B, C)** Effects of IL-23 on capsaicin-induced spontaneous pain in males, as shown by time course **(B)** and accumulated value **(C)**. n = 5 male mice/group. n.s., not significant. **(D, E)** Effects of IL-23 on capsaicin-induced spontaneous pain in females, as shown by time course **(D)** and accumulated value **(E)**. n = 5 female mice/group. *p < 0.05, Mann-Whitney test **(E)**. Data shown as mean ± SEM.

Capsaicin produces acute spontaneous pain, such as licking and lifting of the affected paw, following intraplantar injection in mice (35). We also examined the role of IL-23 in the sex dimorphism of spontaneous pain as induced by capsaicin. In order to see a potentiation by IL-23, we tested a low-dose of capsaicin (500 ng). Intraplantar capsaicin was given 1 h after intrathecal injection of 100 ng of IL-23 or vehicle (PBS) (**Figure 4A**). While intraplantar IL-23 targets macrophages in hind paw skin, intrathecal IL-23 could target macrophages in DRG (27). IL-23 can enhance TRPV1 activity both in DRG neuron cell bodies (mediated by intrathecal injection) and in skin nociceptor terminals (mediated by intraplantar IL-23). We have shown that either intraplantar or intrathecal IL-23 is sufficient to induce mechanical pain in female mice (27). We found that males given vehicle (PBS) and males given IL-23 showed comparable durations of pain response following capsaicin injection (*p* > 0.05, **Figures 4B, C** **)**, while females given IL-23 showed significantly higher durations of pain response than their vehicle counterparts (*p <* 0.05, **Figures 4D, E** **)**. These results suggest that IL-23 potentiates capsaicin-induced spontaneous pain in only females and not in males. Since IL-23 was given by intrathecal injection and IL-23 does not act on nociceptors directly (27), IL-23 may indirectly sensitize TRPV1 at DRG cell bodies to potentiate intraplantar capsaicin-evoked spontaneous pain.

### DRG Macrophages of Both Sexes Express IL-23

**Figure 5:**
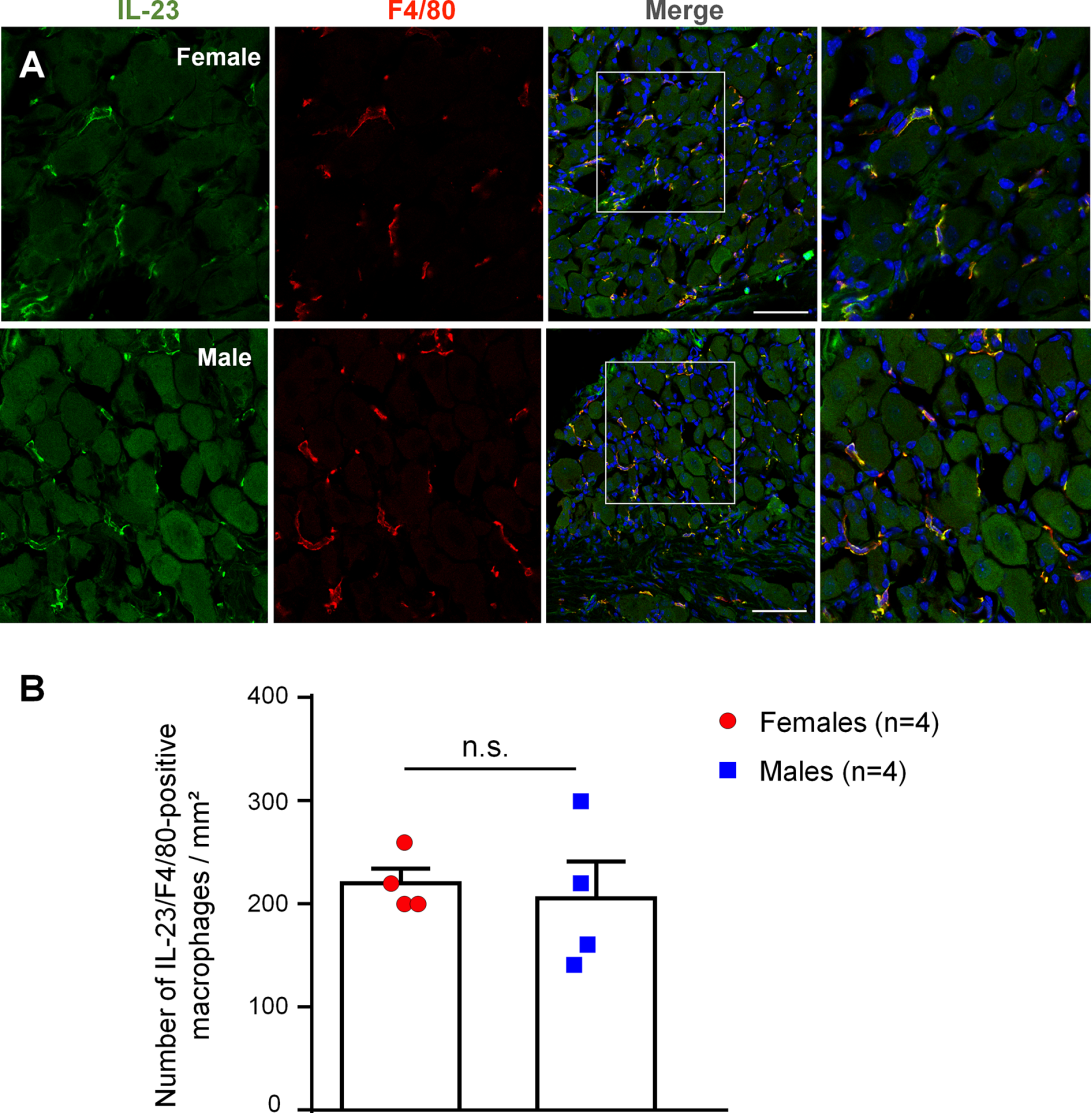
IL-23 is expressed by macrophages in mouse DRG of both sexes. **(A)** Double staining shows co-localization of IL-23 with F4/80. Immunohistochemistry was performed with IL-23 and F4/80 staining on DRG sections of mice to assess IL-23 expression in macrophages. Results were imaged with a Confocal microscope. Columns from left to right represent IL-23 staining (green), F4/80 staining (red), merged images (yellow). The boxes in the third columns are magnified in rightmost images. DRG sections were counter stained with DAPI to label all the nuclei. Scale bars, 75 μm. **(B)** Number of IL-23 and F4/80 double-labeled macrophages in DRG sections of both sexes. Colocalization of IL-23 and F4/80 staining was quantified with Image J and quantification results were used to calculate the number of IL-23/F4/80-positive macrophages per mm^2^. Data were then analyzed in GraphPad Prism using an unpaired t-test. n = 4 mice per sex, and 4 DRG sections were included for each animal. Data shown as mean ± SEM. n.s., not significant.

We used immunohistochemistry to examine IL-23 expression in DRGs of male and female mice. IL-23 immunoreactivity was found in DRG sections of both sexes, and we did not see IL-23 expression in DRG neurons (**Figure 5A**). Double staining revealed a high degree of co-localization of IL-23 and F4/80, a marker for DRG macrophages (Luo et al., 2019b), in both sexes (**Figure 5A**). Further quantitative analysis revealed that females had a slightly higher number of IL-23-positive macrophages than males, albeit not to a significant extent (218.42 ± 14.04/mm^2^ in females *vs*. 203.53 ± 35.68/mm^2^ in males, *p* = 0.7180, **Figure 5B**). These results indicate the presence of IL-23 in DRG macrophages of naïve mice, providing a cellular base for IL-23-mediated macrophage and nociceptor interaction.

### IL-23 Enhances p38 Phosphorylation in DRG Nociceptive Neurons in a Female-Dominant Manner

**Figure 6:**
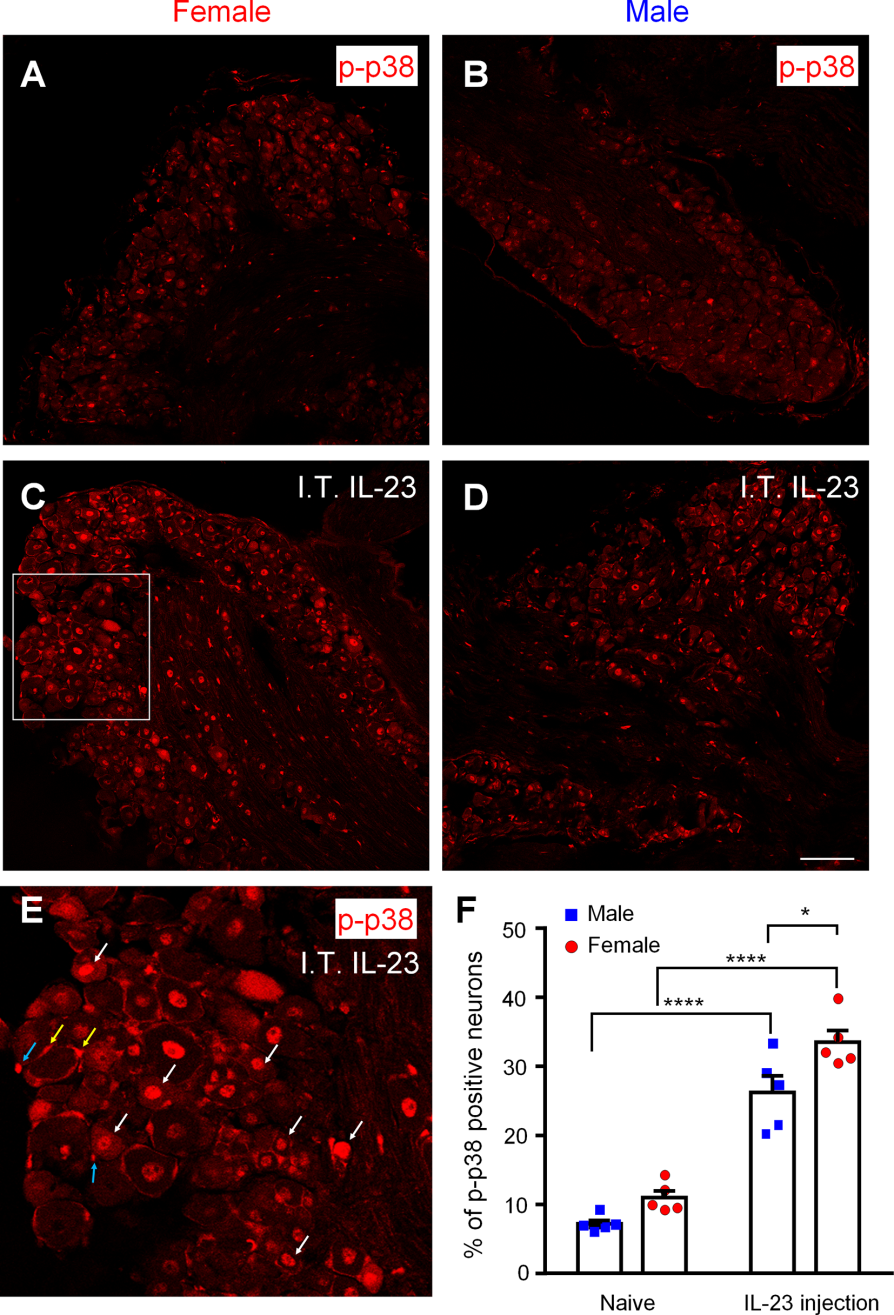
Effects of intrathecal IL-23 treatment on p38 phosphorylation in DRG neurons in male and female mice. **(A–D)** Immunohistochemistry was performed to identify p-p38-positive neurons in the DRG of female mice **(A, C)** and male mice **(B, D)**, without IL-23 treatment **(A, B)** and with IL-23 treatment **(C, D)**. Animals were sacrificed 30 min after intrathecal (I.T.) IL-23 (100 ng) treatment. Scale bar, 100 μm. **(E)** High magnification image of the white box in **(C)**. White arrows indicate activation of many small neurons (presumably nociceptors). Yellow and blue arrows indicate activation of some satellite glial cells and immune cells, respectively. **(F)** Quantification of the percentage of p-p38-positive neurons in mouse DRGs using Image J. Data were then analyzed with GraphPad Prism. n = 5 mice per sex. *p < 0.05, ****p < 0.0001, Two-way ANOVA followed by Bonferroni’s posthoc test. Data shown as mean ± SEM.

Phosphorylation of p38 MAP kinase (p-p38) is a marker for nociceptor activation, as p-p38 is induced in DRG neurons by noxious stimulation, inflammation, and nerve injury and contributes critically to the pathogenesis of pain (36–39). To study the role of IL-23 in pain signaling in DRG neurons, we used immunohistochemistry to stain for p-p38 in the DRG of male and female mice (**Figure 6**), without IL-23 treatment (**Figures 6A, B** **)**, and with IL-23 treatment (**Figures 6C–E**). In support of previous reports (36, 37), p-p38 was primarily present in the nuclei, and the basal levels of p-p38 in DRG neurons were lower in both sexes, labeling 5-15% of DRG neurons in both sexes. Female DRG had a slightly higher basal levels compared to male DRG (p > 0.05, **Figure 6F**). In order to target DRG cells bodies, we injected IL-23 (100 ng) *via* intrathecal (I.T.) route (36, 40, 41). Thirty minutes following IL-23 treatment, p-p38^+^ neurons increased in both sexes (p < 0.0001), labeling 20-40% of DRG neurons in both sexes (**Figure 6F**). However, female DRGs had a significantly higher percentage of p-p38-positive neurons than male DRGs (p < 0.05, **Figure 6F**). After IL-23 treatment, the majority of p-p38-positive cells were small-sized DRG neurons (presumably nociceptors, **Figure 6E**), indicating an activation of nociceptors by IL-23. In addition, we also observed p-p38 expression in the nuclei of non-neuronal cells in female DRG, including satellite glial cells and immune cells (**Figure 6E**). Taken together, these results suggest that IL-23 can promote pain *via* activation of nociceptors in a sex-dependent manner. Since p-p38 is highly co-localized with TRPV1 in DRG neurons under inflammatory conditions and further regulates TRPV1 expression and activity (14, 37), p38 activation may also contribute to IL-23-induced pain.

### Macrophages Release IL-23 and IL-17A

**Figure 7:**
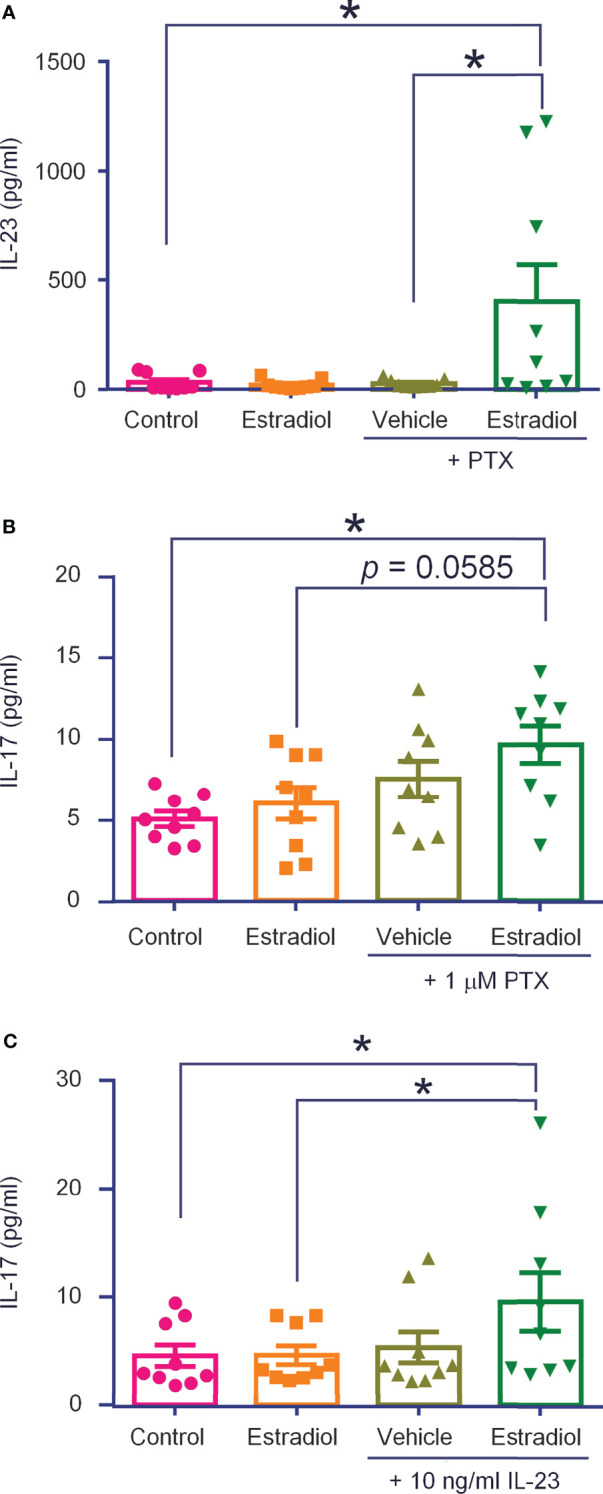
THP-1 macrophages release IL-23 and IL-17A following estrogen and paclitaxel administration. ELISA tests were performed to measure IL-23 **(A)** and IL-17A **(B)** levels produced by THP-1 cells (500,000 in 400 μl), stimulated with estradiol (1 ng/ml, 24 h), paclitaxel (PTX, 1 μg/ml, 24 h), or estradiol plus paclitaxel **(A, B)**, or estradiol and IL-23 [10 ng/ml, 24 h, **(C)**]. n = 9 cultures from two separate experiments. *p < 0.05, One-way ANOVA followed by Bonferroni’s posthoc test. Data are shown as mean ± SEM.

Our recent study showed that IL-23 does not activate nociceptors directly. Instead, IL-23 produces IL-17A in macrophages to activate nociceptors (Luo et al., 2021). To further address the question of IL-23 release and IL-23 signaling in macrophages, we conducted ELISA analysis in THP-1 cells, using a well-characterized cell line of human macrophages. THP-1 cells constitutively released IL-23, with a baseline ranging from 6 to 90 ng/ml from 500,000 cells with 400 μl medium (**Figure 7A**). We stimulated THP-1 cells with estrogen (17β-Estradiol, 1 ng/ml) and paclitaxel (PTX, 1 µg/ml), a chemotherapy drug that can induce neuropathic pain. PTX was also shown to act on peritoneal macrophages to produce inflammatory cytokines (TNF and CXCL1) (42). Application of low dose PTX or estrogen (24 h) did not produce significant increase in IL-23 secretion, but a combination of PTX and estrogen significantly increased IL-23 release in culture medium (*p* < 0.05, *vs* control or PTX alone, **Figure 7A**).

We recently showed that IL-17A was required for IL-23-induced mechanical pain (Luo et al., 2021). At the baseline, THP-1 cells secreted low levels of IL-17A (3-7 ng/ml), but this secretion was significantly increased by co-application of PTX (1 μg/ml, 24 h) and estrogen (1 ng/ml, 24 h), (*p* < 0.05, *vs* control, **Figure 7B**). Interestingly, stimulation with IL-23 (10 ng/mL, 24 h) alone did not increase IL-17A secretion, but co-application of IL-23 (10 ng/ml, 24 h) and estrogen (1 ng/ml, 24 h) did (p < 0.05, *vs*. control and estrogen, **Figure 7C**). Considered together, these results suggest (1) THP-1 macrophages secrete IL-23 and IL-17A and (2) this secretion is increased by estrogen and chemotherapy. These results also suggest IL-17A may be downstream of IL-23 signaling in macrophages.

## Discussion

In this study, we confirmed our recent finding that IL-23 evoked pain in female mice but not male mice (27) by a different investigator. Notably, IL-23 evoked mechanical, but not thermal heat or cold pain (27), indicating that IL-23-mediated sex dimorphism of pain is modality specific. Further, we found that TRPV1-expressing nociceptors and TRPV1 protein were critically required for IL-23-induced mechanical pain (27). However, it has remained unclear if IL-23 also potentiates spontaneous pain in a TRPV1- and sex-dependent manner. In this study, we assessed spontaneous pain using two different approaches: an optogenetic approach to activate TRPV1-expressing nociceptors and a pharmacological approach to activate TRPV1 using capsaicin. Our data demonstrate that IL-23 potentiates both types of C-fiber-dependent spontaneous pain, induced by blue light and capsaicin, in female but not male mice.

One of the interesting findings of this study is the demonstration of sexual dimorphism in a subset of *Trpv1* ^+^ neurons with very small sizes (100-200 µm^2^). Although capsaicin evoked mechanical allodynia in both sexes, only female mice exhibited decreased withdrawal thresholds to low-dose capsaicin (50 ng) (27). This is in line with results from a published clinical study showing that topical administration of capsaicin results in higher pain intensity and unpleasantness in women than men (43). Our finding is also in line with results from several pre-clinical studies showing that TRPV1 signaling is mediated by sex hormones. Estrogen was shown to sensitize TRPV1 in dissociated sensory neurons and potentiate TRPV1-mediated mechanical pain (44). Prolactin modulates TRPV1 activity in female sensory neurons in an estrogen-dependent manner (45) and produces female-specific pain (46, 47). A recent study also demonstrated sex differences in nociceptor translatomes (48). However, we and others did not see significant sex differences in overall expression of TRPV1 in mouse and human DRGs (27, 48). Although it is well-established that TRPV1 is expressed by C-fiber neurons, our results suggest that there are different populations of TRPV1^+^ nociceptors and only a subset of TRPV1^+^ neurons with very small sizes (100-200 µm^2^) show sex dimorphism. Consistently, estrogen receptor-alpha (Erα) is co-expressed with TRPV1, and selective deletion of Erα in TRPV1^+^ nociceptors abolished IL-23-evoked mechanical pain in females. It will be of great interest to investigate whether this subset of TRPV1^+^ nociceptors (100-200 µm^2^) has greater response to estrogen.

p38 MAPK plays an important role in the pathogenesis of inflammatory pain and neuropathic pain (49). Notably, p38 activation in spinal cord microglia regulates neuropathic pain in male but not female mice (50–53). In contrast, p38 could be activated in primary sensory neurons of both sexes, and IL-23 increases neuronal p-p38 levels in both sexes, with a greater increase in females. Thus, different cell types may have distinct sex dependence, even for the same signaling pathway. Both TRPV1 expression and p38 activation (p-p38) in nociceptors are upregulated by inflammation with specific p-p38 induction in TRPV1+ neurons, and furthermore, p38 activation is necessary for inflammation-induced TRPV1 upregulation in nociceptors (37). p38 MAPK appears to be an important link between cytokines and nociceptor activation. TNF-α and IL-1β have been shown to activate p38 in nociceptors, and furthermore, p38 inhibitor can block nociceptor sensitization by these cytokines (13, 14, 54). It is of great interest to investigate how IL-23 activates p38 in nociceptors. Unlike TNF-α and IL-1β, IL-23 does not cause direct activation of nociceptors, as shown by calcium imaging and electrophysiology studies (27), but also see different result in another report (21). Instead, flow cytometry revealed IL-23R expression in DRG and peritoneal macrophages. Our histochemical data showed that IL-23 primarily expresses by mouse macrophages in DRG of both sexes. Importantly, we also showed that THP-1 cells (a human macrophage cell line) could produce IL-23, and IL-23 release increased in response to simultaneous stimulation of estrogen and the chemotherapy drug paclitaxel (**Figure 7C**). We also revealed IL-17A as a downstream signaling event of IL-23 in macrophages. Thus, incubation of THP-1 cells with a combination of estrogen and IL-23 increased IL-17A secretion. Consistently, IL-23 caused IL-17A release in mouse peritoneal and DRG macrophages (27). Importantly, IL-17A receptor (IL-17AR) but not IL-23R is expressed by nociceptors; and female nociceptors exhibit greater sensitivity to IL-17A in both mice and nonhuman primates (27). While our study focused on IL-23 in macrophage-nociceptor signaling, nociceptor-dendritic cell-T cell signaling has been shown to activate the local IL-23/IL-17 cascade in the skin *via* CGRP release in psoriasis (19, 22). Furthermore, TRPV1+ neuron activation is sufficient to elicit host defense against infections through local nerve reflex (25). Future studies are warranted to determine distinct contributions of the IL-23/1L-17 cascade in different immune cells (macrophages *vs*. dendritic/T cells) under different disease conditions.

In summary, emerging evidence suggests that neuro-immune interactions play an important role in the sexual dimorphism of pain (55, 56). Macrophages may promote pain in both sexes through distinct signaling mechanisms. For example, macrophage Toll-like receptor 9 signaling drives TNF-α and CXCL1 release and evoke mechanical pain in male mice (42), whereas macrophage IL-23/IL-17 signaling regulates mechanical pain in females (27). The present study demonstrated the role of IL-23 in inducing spontaneous pain in females and, further explored the IL-23-mediated macrophage-nociceptor interaction using optogenetic and biochemical approaches. Optogenetics offers a unique way to modulate nociceptor activity, compared to traditional thermal, mechanical, and chemical stimuli. In future studies we will further investigate how light activation of nociceptors would alter cytokine release from immune cells and to determine the nature of sex-specific cytokine-nociceptor signaling in pain.

## Data Availability Statement

The original contributions presented in the study are included in the article/supplementary material. Further inquiries can be directed to the corresponding author.

## Ethics Statement

The animal study was reviewed and approved by Duke University IACUC.

## Author Contributions

JJ did optogenetic, behavioral, and RNAscope experiments and analyzed data. QH conducted histochemical experiments. XL conducted behavioral experiments. SB conducted ELISA experiments. YM assessed paw edema. AM helped with histology. JJ and R-RJ wrote the paper. AGN contributed to project discussion and edited the paper. All authors contributed to the article and approved the submitted version.

## Funding

This work was supported by Duke University Anesthesiology Research Fund.

## Conflict of Interest

The authors declare that the research was conducted in the absence of any commercial or financial relationships that could be construed as a potential conflict of interest.

## Publisher’s Note

All claims expressed in this article are solely those of the authors and do not necessarily represent those of their affiliated organizations, or those of the publisher, the editors and the reviewers. Any product that may be evaluated in this article, or claim that may be made by its manufacturer, is not guaranteed or endorsed by the publisher.
